# Prevalence and characteristics of coronary arteritis within a prospective observational cohort of patients with Takayasu’s arteritis

**DOI:** 10.3389/fimmu.2026.1768422

**Published:** 2026-03-16

**Authors:** Safa Farrukh, Kaitlin A. Quinn, Alessandra Brofferio, Kathleen Mitchell, W. Patricia Bandettini, Bhanu Richa Duggirala, Michael Ring, Marcus Y. Chen, Peter C. Grayson

**Affiliations:** 1 National Institute of Arthritis and Musculoskeletal Diseases, National Institutes of Health (NIH), Bethesda, MD, United States; 2 National Heart, Lung, and Blood Institute, National Institutes of Health (NIH), Bethesda, MD, United States; 3 Biomedical Translational Research Informatics (BTRIS), National Institutes of Health (NIH) Clinical Center, National Institutes of Health, Bethesda, MD, United States

**Keywords:** angiography, computed tomography, coronary artery, Takayasu’s arteritis, vasculitis

## Abstract

**Introduction:**

Due to the rarity of the condition, optimal assessment and therapeutic strategies to manage coronary arteritis in Takayasu’s arteritis (TAK) have not been well defined.

**Methods:**

Cases of coronary arteritis were identified within an ongoing single-center prospective observational cohort study in TAK. Patients underwent standardized clinical, imaging, and laboratory assessment per protocol with centralized review of data. Imaging assessment included non-invasive angiography of the aorta and branch vessels, cardiac computed tomographic angiography, cardiovascular magnetic resonance imaging, and positron emission tomography (PET). Cardiac involvement was defined based on demonstration of at least one vasculitic lesion within a coronary artery by an appropriate imaging study.

**Results:**

The prevalence of coronary arteritis was 13 (9%) out of 137 patients with TAK. Patients with and without coronary arteritis were similar in terms of demographics, angiographic pattern of disease, and non-cardiac clinical symptoms. Vasculitic lesions typically were stenosing and involved the proximal coronary arteries. Active vasculitis by PET in the ascending aorta was associated with active coronary arteritis (sensitivity 100%, specificity 67%). Favorable clinical outcomes were generally achievable but often required medical therapy and vascular intervention. Anti-cytokine medical therapies were likely more effective than cytotoxic therapies. Fifty percent of patients had complications from vascular grafts or stents, respectively, often prompting additional vascular procedures.

**Discussion:**

Coronary arteritis is an uncommon complication in TAK. Multimodal imaging can be useful to diagnose, monitor, and manage coronary arteritis. While medical therapy is preferred, vascular intervention may be necessary, and complications from attempts at vascular reperfusion are common.

## Introduction

Takayasu’s arteritis (TAK) is a rare but life-threatening large vessel vasculitis that primarily affects children and younger adults (1). The disease is defined by vasculitic involvement of the aorta and its primary branches with resulting luminal damage. Cardiac involvement from coronary arteritis is uncommon in TAK but can have devastating consequences (2). Major cardiovascular events are an important cause of mortality in TAK (2). Myocardial infarction is estimated to occur in 3-24% of patients (2). Identification of patients with TAK at increased risk for cardiac events, detailing the clinical features and imaging characteristics of coronary arteritis, and defining optimal medical and surgical approaches, is an unmet need.

Because coronary arteritis is an uncommon feature in what is already a rare disease, there is a paucity of data to guide clinical assessment and management in these scenarios. Most published information about vasculitic involvement of the coronary arteries in TAK is limited to case reports or small case series (3–5, 21, 22). There are few prospective observational cohorts to detail the characteristics and prevalence of coronary arteritis and report long term outcomes (6, 23). Different patterns of coronary artery involvement have been described: arteritis of the proximal coronary arteries, focal lesions in the more distal aspects of the coronary vasculature, and diffuse coronary disease (2). Resulting stenoses or aneurysms have been reported (2). Consensus guidelines regarding the management of TAK were recently published; however, there was no direct mention about management of coronary arteritis in these patients due to a lack of supporting evidence (7). When to pursue medical versus surgical treatment, which medications to use preferentially, and optimal interventional strategies are unknown.

The goals of this study were to detail the clinical and imaging aspects of coronary arteritis in association with disease presentation and long-term outcomes using data collected within an ongoing prospective observational cohort in TAK.

## Materials and methods

### Study design and clinical assessment

Patients with TAK who met the 2022 ACR/EULAR Classification Criteria for TAK (15) were selected from an ongoing prospective, observational cohort study of systemic vasculitis at the National Institutes of Health (14-AR-0200). This study was approved by a local institutional review board, and written informed consent was obtained from each participant. Patients were evaluated for a baseline study visit at any point in the disease course and were directly evaluated at 6–12 month intervals, whenever feasible. Standardized data collection forms were utilized at all study visits to record clinical aspects of disease (24). All available outside clinical records and imaging studies prior to study entry were obtained for direct review.

### Assessment of cardiac involvement

Patients were classified as having cardiac involvement based on demonstration of at least one vasculitic lesion within a coronary artery upon direct review of an appropriate imaging study by the study investigative team. In addition to standardized clinical assessment, a detailed cardiac history was obtained for each patient with cardiac involvement. Patients were questioned about timing of onset of cardiac disease relative to diagnosis, type of cardiac symptoms, medical and surgical interventions, and outcomes. Chart review was performed using all available outside records with focus on development of a Non-ST-Elevation Myocardial Infarction (NSTEMI) or a ST-Elevation Myocardial Infarction (STEMI), type of interventions performed (grafts, percutaneous coronary interventions, or angioplasty), complications of interventions (presence of in-stent restenosis or graft occlusion), and clinical outcomes in response to specific medical therapies or interventions.

### Vascular imaging of the aorta and branch arteries

Each patient underwent non-invasive angiography at study entry with either magnetic resonance or computed tomographic imaging of the large vasculature from the carotid bifurcation to the mid-thigh region. The scans were assessed for luminal damage (occlusion, stenosis, aneurysm) in the following territories: intracranial, right coronary, left coronary, right carotid, left carotid, right subclavian, left subclavian, right axillary, left axillary, thoracic aorta, ascending aorta, descending aorta, abdominal aorta, mesenteric, right iliofemoral, left iliofemoral, right renal, and left renal arteries.

At the baseline visit, each patient also underwent FDG-positron emission tomography (PET). FDG-PET computed tomography (CT) was performed in adult patients, and FDG-PET magnetic resonance (MR) imaging was performed in patients <18 years of age. Arterial FDG uptake in specific territories, corresponding to angiographic assessment, was determined by visual inspection relative to the liver by two independent readers, blinded to clinical status, with adjudication of differences by consensus. FDG uptake greater than the liver in a territory was considered evidence of active vasculitis.

Angiography and PET imaging were repeated at future study visits at the discretion of the study investigators.

### Cardiac imaging and interpretation

Patients with known coronary artery disease by review of outside records or suspected coronary artery disease based upon direct clinical assessment underwent cardiac computed tomography angiography (CTA) imaging whenever feasible. Patients who had ongoing angina or confirmed coronary artery lesions by CTA with suspicion for active disease underwent cardiovascular magnetic resonance (CMR) with stress testing.

### Cardiac CT

ECG-gated CTA was performed on a 320-detector row scanner (Aquilion ONE, Canon Medical, Otawara, Japan). Oral and/or intravenous metoprolol was administered to achieve a target resting heart rate <60 beats/min. Nitroglycerin vasodilated CTA images were acquired after intermittent bolus tracking of iopamidol-370 (Bracco Diagnostics, Princeton, NJ, USA) radiocontrast (1–1.5 mL/kg) in the descending aorta. Images were reconstructed with 0.5 mm slice thickness and 0.25 mm increment and interpreted on a dedicated workstation (Vitrea, Vital Images, Minnetonka, MN, USA) by a clinical radiologist. Interpretations followed published guidelines (8). The exact location of each coronary lesion was noted based on standard coronary artery anatomy (9). The type of lesion was classified as a vasculitic or atherosclerotic lesion. The presence of vasculitic coronary artery lesion(s) was defined by a coronary artery lesion that had inflammatory characteristics such as fat stranding and wall thickening. An atherosclerotic coronary lesion was defined as a well delineated coronary plaque (with or without areas of calcification) causing luminal narrowing without fat stranding surrounding the vessel.

Other characteristics of the lesions, such as the length and width of the coronary artery lesion, degree of stenosis, and presence of calcification, were assessed by a central reader. Degree of stenosis was categorized as the following based on the Society of Cardiovascular Computed Tomography (SCCT) stenosis grading system: none, < 25%, 25-49%, 50-69%, >70%, or 100% (occluded) (10). Coronary artery wall thickness was assessed on CTA scan and if the thickness was measured as abnormal (>eq 1mm), the width of the wall was recorded (11). Agatston calcium score was recorded (12). Grading for coronary artery disease was done based on the degree of luminal narrowing. Prior percutaneous coronary interventions, location of the stent, and evidence of in-stent restenosis in the coronary arteries were assessed on the CTA scan. Aortic root thickness was assessed on CTA scan, and if the aortic thickness was found to be abnormal (>2mm), the width of the aortic root was measured and noted (11).

### Cardiovascular magnetic resonance

Cardiovascular magnetic resonance studies were performed at 1.5 T field strength (Aera, Siemens, Erlangen, Germany) with standard cine images of cardiac function and standard late gadolinium enhancement (LGE) images for viability/scar assessment after administration of gadobutrol 0.15 mmol/kg (Gadavist Bayer Pharma AG, Leverkusen, Germany). Interpretation was performed visually by a level 3 CMR cardiologist using a dedicated workstation (Leonardo, Siemens, Erlangen, Germany) or software (SuiteHEART, NeoSoft, Pewaukee, WI). CMR reports were interpreted based on the Society for Cardiovascular Magnetic Resonance (SCMR) guidelines (13).

CMR reports were reviewed and viability, rest ventricular volumes and function, rest perfusion, and stress perfusion data were aggregated using standardized case report forms. First, late gadolinium enhancement (LGE) was categorized as normal or abnormal for each patient. If LGE was abnormal in an infarction pattern, the territory affected and degree of late gadolinium enhancement (LGE) was listed. An abnormality in LGE imaging may correlate with infarction but can also be abnormal in other conditions such as non-ischemic dilated cardiomyopathy, myocarditis, or infiltrative processes. Abnormal viability was identified as a pattern consistent with CAD infarction, classified by degree of LGE with the following categories: 1-25, 26-50, 51-75, 76-99, 100 per cent transmural extent of enhancement. If rest function was abnormal, the territory affected and the wall motion abnormality was recorded. Rest function indicates contractile function of the heart walls under resting conditions and demonstrates the presence of wall motion abnormalities including akinesis, hypokinesis, dyskinesis, and aneurysm. The 17-segment Left Ventricle Model with Coronary Distributions was used to record the left ventricle myocardial territory supplied by the coronary artery affected by abnormal viability and rest function defects (14). Data collected on CTA and CMR findings were compared for each patient.

### Statistical analysis

Clinical characteristics at study entry were compared between TAK patients with and without coronary involvement, including demographics, diagnostic delay, clinical symptoms, medication use, comorbidities, laboratory values, and vascular patterns of disease involvement. Diagnostic delay was defined as the time interval between symptom onset attributable to vasculitis and date of diagnosis. Continuous variables were compared using Mann-Whitney tests and categorical variables were compared using a two-tailed Fisher’s exact test. Confidence intervals were calculated using Clopper-Pearson exact method. All analyses were performed using JMP Version 18. Adjustment for multiple comparisons was not performed due to small sample size, and a p-value <0.05 defined statistical significance.

## Results

### Study participants

Data was collected from 137 patients with TAK. The demographics of the cohort were consistent with clinical expectations (**Table 1**). The median age at symptom onset was 23 years, with 49 (36%) patients presenting at <18 years of age. The median disease duration from the time of symptom onset to the baseline study visit was 4 years. Fifty percent of the cohort experienced relapsing disease, and most patients had been treated with a conventional disease-modifying anti-rheumatic drug (DMARD) (82%) or biologic DMARD (55%).

**Table 1:** Baseline characteristics of study participants.

Variable	Total cohort N=137	Patients with coronary arteritis N=13	Patients without coronary arteritis N=124	P-value
Age (median, interquartile range)				
At baseline study visit	33 (21-40)	34 (18-39.5)	33 (20-40)	0.80
At symptom onset	23 (15-33)	23 (15-34)	23 (14-32)	0.84
At diagnosis	26 (16-36)	26 (16-36)	25 (15-33)	0.47
Disease Duration (years)	4 (1-13)	6 (1-12)	4 (1-13)	0.56
Sex (n, % Female)	117 (85%)	13 (100%)	104 (84%)	0.21
Race (n, % White)	94 (69%)	10 (77%)	84 (68%)	1.00
Ethnicity Latino or Hispanic	18 (13%)	2 (15%)	16 (13%)	1.00
Complications				
Cerebrovascular accident	18 (13%)	0 (0)	18 (15%)	0.22
Heart valve replacement	9 (7%)	1 (8%)	8 (6%)	1.00
Pulmonary hypertension	2 (1%)	0 (0%)	2 (2%)	1.00
Thromboembolic disease	7 (5%)	0 (0%)	7 (6%)	1.00
Vascular Intervention	55 (40%)	9 (69%)	46 (37%)	0.04
Relapse	69 (50%)	6 (46%)	63 (51%)	0.75
Treatment				
Conventional DMARD ever	111 (82%)	10 (77%)	101 (82%)	0.71
Biologic DMARD ever	74 (55%)	7 (58%)	67 (54%)	1.00
>1 Biologic DMARD	25 (19%)	4 (33%)	21 (17%)	0.23
Lab results				
Elevated APR ever	106 (23%)	10 (77%)	96 (77%)	1.00
Elevated APR time of diagnosis	98 (72%)	10 (77%)	88 (72%)	1.00

DMARD, disease modifying anti-rheumatic drug, APR, acute phase reactant.

### Prevalence of coronary arteritis and associated clinical characteristics

The prevalence of coronary arteritis was 13 (9%) out of 137 patients with TAK. All patients with coronary arteritis were female. Comparing between patients with or without a history of coronary arteritis, there were no significant differences with respect to age at disease onset, age at diagnosis, sex, race, ethnicity, history of relapsing disease, treatment, or history of elevated acute phase reactants. Patients with coronary arteritis were more likely than those without coronary arteritis to undergo vascular intervention of any kind (69% *vs* 37%, p=0.04).

Specific clinical symptoms are listed in **Table 2**. There were few significant differences in clinical features between patients with and without coronary arteritis. Compared to patients without a history of coronary arteritis, fewer patients with coronary arteritis had a history of frontotemporal headaches (0% *vs* 31%, p=0.04) and more patients had a history of abdominal pain (39% *vs* 15%, p=0.05).

**Table 2:** Clinical symptom prevalence at any point in disease in patients with and without coronary vasculitis.

Clinical symptom	Coronary arteritis N=13	No coronary arteritis N=124	P-value
Fever >38C	1 (8%)	27 (22%)	0.47
Weight loss (≥5kg)	2 (15%)	34 (27%)	0.51
Fatigue	7 (54%)	83 (66%)	0.36
Vertigo	0 (0%)	8 (6%)	1.00
Lightheadedness	1 (8%)	23 (19%)	0.46
Positional lightheadedness	3 (23%)	52 (42%)	0.24
Generalized headache	4 (31%)	33 (26%)	0.75
Right frontotemporal headache	0 (0%)	31 (25%)	0.04
Left frontotemporal headache	0 (0%)	31 (25%)	0.04
Right posterior headache	1 (8%)	9 (7%)	1.00
Left posterior headache	1 (8%)	12 (10%)	1.00
Blurred vision	0 (0%)	25 (24%)	0.13
Right vision loss	1 (8%)	9 (7%)	1.00
Left vision loss	0 (0%)	11 (9%)	0.60
Amaurosis Fugax	1 (8%)	8 (6%)	1.00
Right jaw claudication	1 (8%)	10 (8%)	1.00
Left jaw claudication	1 (8%)	11 (9%)	1.00
Right carotidynia	1 (8%)	25 (20%)	0.46
Left carotidynia	3 (23%)	32 (26%)	1.00
Right arm claudication	3 (23%)	54 (44%)	0.24
Left arm claudication	4 (31%)	67 (54%)	0.15
Subclavian steal syndrome	0 (0%)	9 (7%)	0.60
Abdominal pain	5 (39%)	19 (15%)	0.05
Renal hypertension (>140/90)	2 (15%)	31 (25%)	0.73
Right leg claudication	3 (23%)	25 20%)	0.73
Left leg claudication	2 (15%)	26 (21%)	1.00

### Clinical description of coronary arteritis

Coronary arteritis was detected at the time of diagnosis in 10 of 13 TAK patients with coronary artery involvement (77%). Eleven of 13 (85%) patients complained of chest pain prompting cardiac evaluation, and coronary disease was incidentally detected by imaging in absence of symptoms in the remaining 2 patients. Myocardial infarction occurred in 7 (54%) patients: 5 patients with STEMI and 2 patients with NSTEMI. No deaths occurred after a median follow up of 6 years.

**Figure 1:**
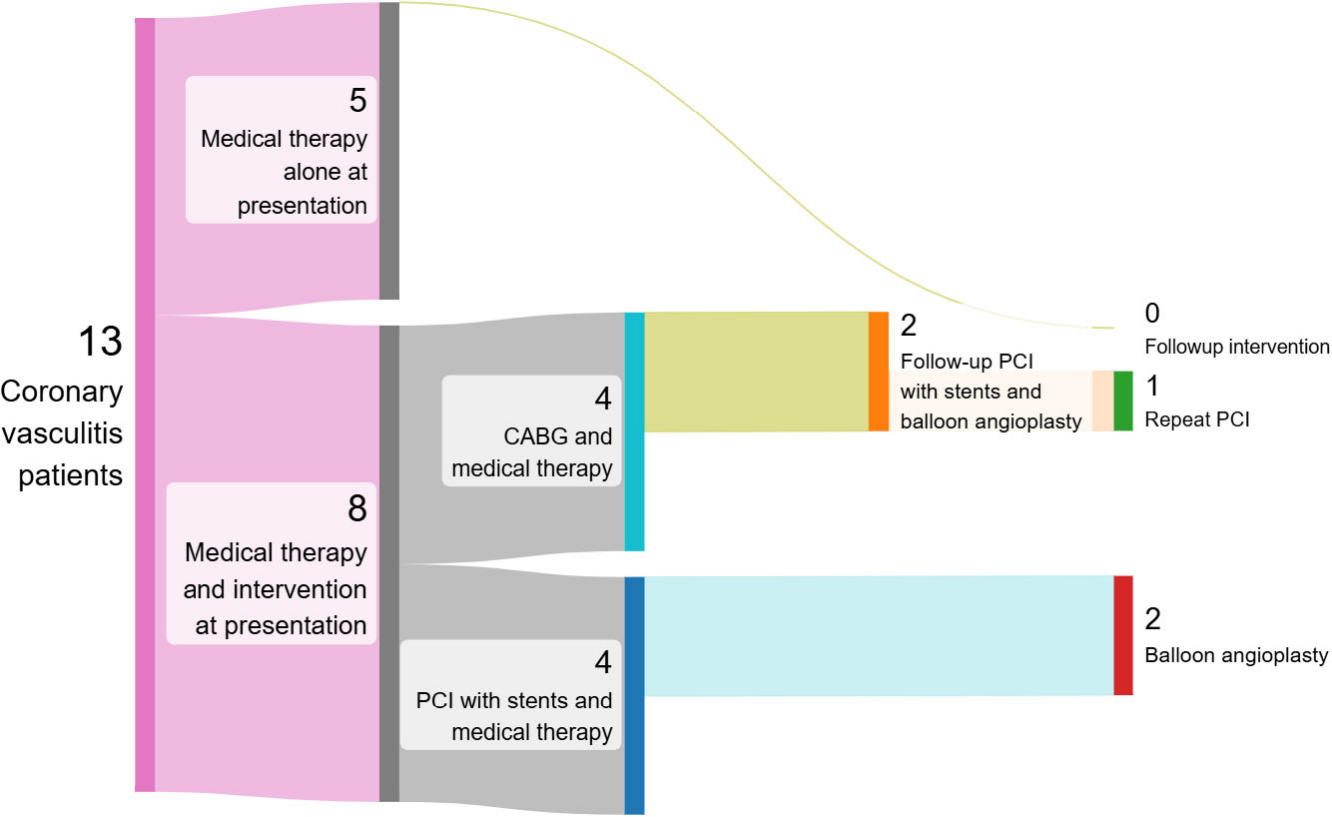
Sankey diagram of therapeutic intervention. Among 13 patients with Takayasu’s arteritis and coronary arteritis, 5 patients were treated with medical therapy alone and 8 patients underwent vascular intervention in addition to medical therapy. Coronary artery bypass graft (CABG) or percutaneous coronary intervention (PCI) was performed in 4 patients respectively and complications were seen in 2 patients in each respective intervention group.

Vascular intervention was performed in 8 patients (62%), all of whom received concomitant medical therapy (**Figure 1**). In 7 out of these 8 patients, vascular intervention was performed in the setting of myocardial infarction. Coronary artery bypass graft (CABG) was chosen as first line vascular intervention in 4 patients, and percutaneous coronary intervention (PCI) with drug-eluting stents was the first line approach in the other 4 patients. Four out of 8 (50%) patients only needed one vascular intervention to achieve reperfusion. Two patients who underwent CABG required a follow up PCI with stents and balloon angioplasty for occluded grafts or stenoses at the surgical anastomosis site. Two patients who received PCI with stents as first line intervention underwent subsequent balloon angioplasty for in-stent restenosis. The average number of stents placed for patients who underwent PCI was 1.2 stents. The average number of grafts placed for patients who underwent CABG was 1.25 grafts.

Five patients (39%) did not undergo vascular intervention for coronary arteritis. TAK was diagnosed incidentally in one of these patients when vascular imaging confirmed abnormal vascular findings detected during a routine physical examination, and this patient never received medication for TAK. Four (31%) patients were treated with medical therapy alone. Tocilizumab and infliximab were the two most commonly used biologic medications. Four out of six patients responded well to tocilizumab, including two patients who previously failed a tumor necrosis inhibitor (TNF) inhibitor. Six out of nine patients responded well to a TNF inhibitor, including one patient who previously failed tocilizumab. Two patients with active coronary arteritis failed to respond to treatment with cyclophosphamide based on persistent clinical symptoms with associated vascular inflammation by FDG-PET but responded to anti-cytokine therapy.

### Cardiac computed tomography

**Figure 2:**
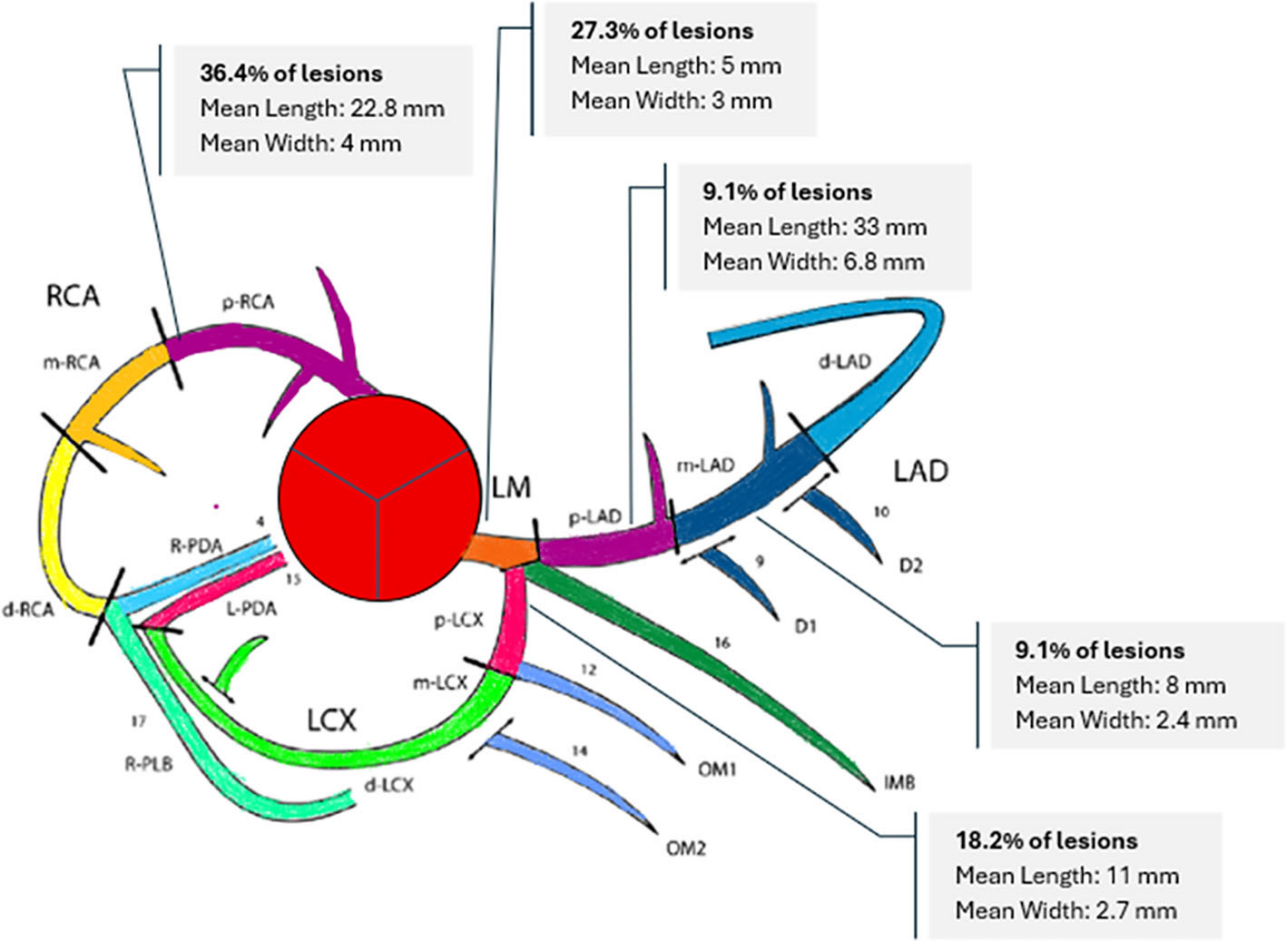
Distribution and characteristics of coronary artery vasculitic lesions in Takayasu’s arteritis cohort. Areas of coronary arteritis tended to occur in proximal portions of the right, left main, or left coronary arteries or in the proximal circumflex or left anterior descending arteries. (LM, left main; LAD, left anterior descending; LCX, left circumflex; RCA, right coronary artery; IMB, intermedius branch; D, diagonal, OM, obtuse marginal; PDA, posterior descending artery; PLB, posterolateral branch; p, proximal; m, mid; d, distal; R, right; L, left).

**Figure 3:**
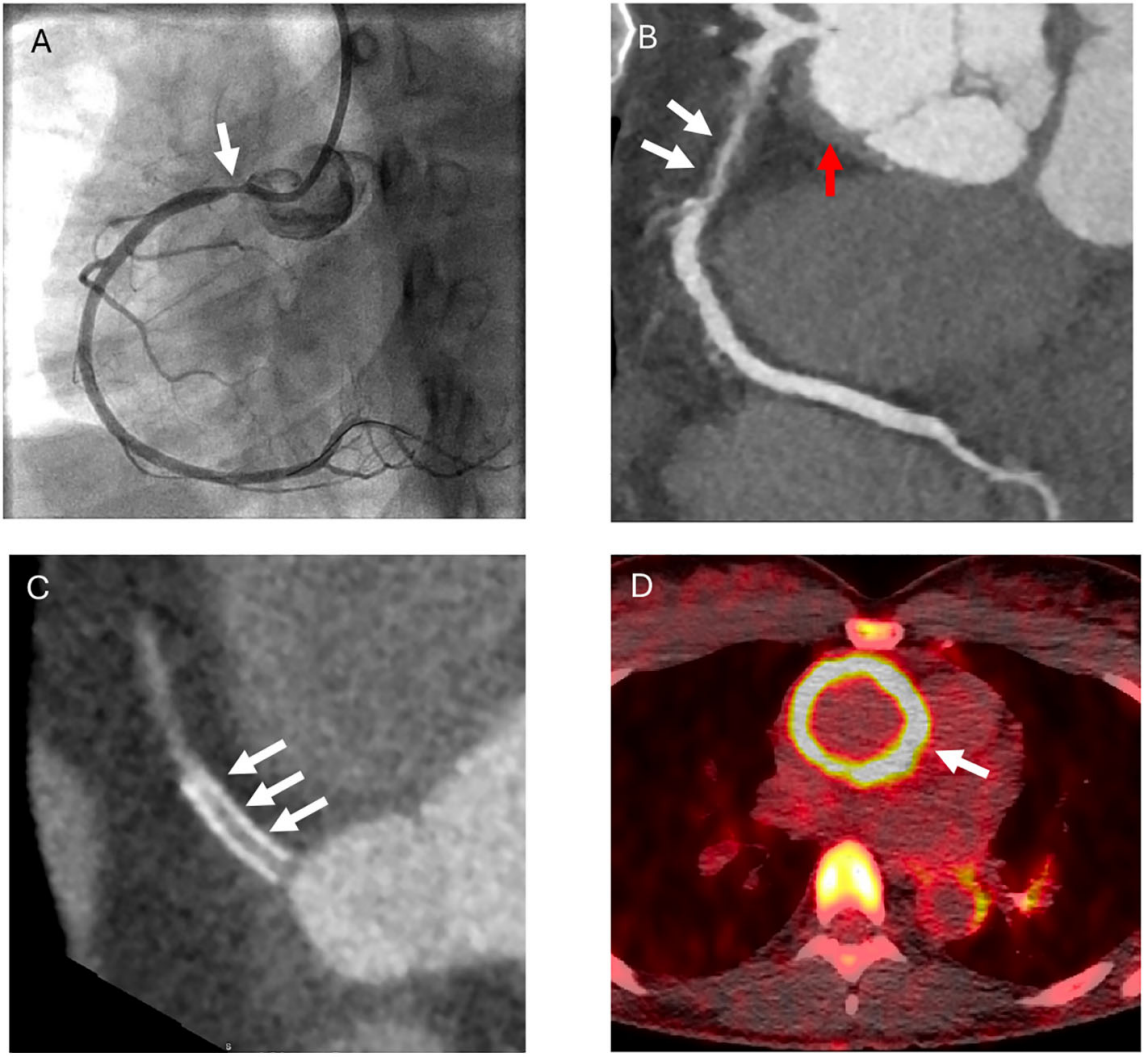
Representative images of coronary artery vasculitis in Takayasu’s arteritis. Catheter based angiography demonstrating ostial vasculitic lesion in the right coronary artery **(A)**. Cardiac computed tomographic angiography showing stenotic proximal right coronary artery with associated wall thickness **(white arrows)** and wall thickness of the aorta (red arrow) **(B)**. Cardiac computed tomographic angiography showing in-stent restenosis in the right coronary artery **(C)**. Positron emission tomography/computed tomography showing increased FDG radiotracer in the ascending aorta in a patient with concomitant active coronary arteritis **(D)**. .

Cardiac CTA was performed at the NIH on eight of the 13 patients with a history of coronary arteritis. Median age at the time of imaging was 35.5 years. Five patients had vasculitic involvement of one coronary artery, two patients had two affected coronary arteries, and one patient had three affected coronary arteries. Number of affected arteries was as follows: proximal right coronary artery = 4; left main coronary = 3; proximal left circumflex=2; proximal left anterior descending = 1; and mid left anterior descending = 1 (**Figure 2**). All lesions in the proximal right or left main coronary arteries resulted in > 50% stenosis, and vascular intervention was performed to correct 6 of the 7 lesions. The median length of a vasculitic coronary lesion was 7 mm and the median width was 3 mm. Associated calcification of the coronary arteries was not present. Aortic root thickening was present in all patients. Three patients with coronary artery stenting all had evidence of in-stent restenosis. Out of the four patients who had coronary artery bypass grafts, two had occluded grafts. Representative images are shown in **Figure 3**.

### Cardiovascular magnetic resonance imaging

Five patients underwent CMR. Three patients with a known history of coronary arteritis were imaged due to clinical concern for ongoing active vasculitis. All three patients had resting function abnormalities in areas corresponding to known coronary artery involvement. Two of these patients, both of whom previously underwent vascular intervention, had abnormal stress perfusion. These patients were both treated with escalated immunosuppressive therapy alone with subsequent clinical improvement. One patient, who had never received vascular intervention, had abnormal rest function but no evidence of stress perfusion abnormalities. This patient was managed with continued medical therapy without further medication changes and did well. One patient underwent CMR to evaluate atypical chest pain and did not have any viability or stress perfusion abnormalities. This patient did well with no changes in medical management. One patient underwent CMR as part of a pre-operative evaluation for a major reconstruction surgery of a severely damaged aorta. The CMR showed a single-vessel myocardial infarction without stress perfusion abnormalities, and no medication changes were made. The patient did well in surgery without any cardiac complications.

### Distribution of angiographic lesions in the aorta and branch arteries

The distribution of arterial lesions across the cohort mirrored known prevalence of disease from other published cohort studies in TAK (**Table 3**). The left subclavian and left carotid arteries were the most common sites of arterial damage. There were no significant differences in the global distribution of arterial disease between patients with and without a history of coronary arteritis. Compared to patients without coronary arteritis, fewer patients with coronary arteritis had right subclavian artery disease (8% *vs* 35%, p=0.06) and more patients had mesenteric disease (38% *vs* 19%, p=0.14) or thoracic aorta disease (69% *vs* 46%, p=0.15). Patients with a history of coronary arteritis had a similar number of affected arterial territories (median = 3, interquartile range 2-5) compared to patients without a history of coronary arteritis (median =4, interquartile range 2 = 7) (p=0.34). Among patients with a history of coronary arteritis, there was no obvious typical pattern of arterial involvement, and the most frequent Numano classification was type V (n=5, 38%) or type 2a (n=3, 23%) (16).

**Table 3:** Comparison of arterial territory affected by vasculitis based on angiography between coronary arteritis and non-coronary arteritis patients.

Arterial territory involved by angiography	Total cohort N=137	Coronary arteritis N=13	No Coronary arteritis N=124	P value
Intracranial	12 (9%)	0 (0%)	12 (10%)	0.60
Right carotid	52 (40%)	2 (15%)	50 (40%)	0.13
Left carotid	75 (54%)	5 (38%)	70 (56%)	0.25
Right subclavian	44 (32%)	1 (8%)	43 (35%)	0.06
Left subclavian	76 (55%)	5 (38%)	71 (57%)	0.25
Right axillary	15 (11%)	0 (0%)	15 (12%)	0.36
Left axillary	16 (12%)	1 (8%)	15 (11%)	1.00
Thoracic aorta	66 (48%)	9 (69%)	57 (46%)	0.15
Ascending aorta	45 (33%)	6 (46%)	39 (31%)	0.35
Abdominal aorta	46 (34%)	6 (46%)	40 (32%)	0.36
Mesenteric	28 (20%)	5 (38%)	23 (19%)	0.14
Right iliofemoral	15 (11%)	2 (15%)	13 (10%)	0.64
Left iliofemoral	15 (11%)	1 (8%)	14 (11%)	1.00
Right renal artery	29 (21%)	2 (15%)	27 (22%)	0.74
Left renal artery	26 (19%)	3 (23%)	23 (19%)	0.71

### FDG-PET involvement and association with active coronary arteritis

Out of 137 patients, 130 (95%) had an FDG-PET scan performed at the baseline study visit. There were few significant differences in FDG-PET uptake in arterial territories between patients with and without coronary arteritis (**Table 4**). Compared to patients with a history of coronary arteritis, more patients with coronary arteritis had FDG-PET activity in ascending aorta (58% *vs* 31%, p=0.06) and fewer patients had FDG-PET activity in the left carotid artery (0% *vs* 30%, p=0.02) and right carotid artery (0% *vs* 26%, p=0.06).

**Table 4:** Comparison of arterial territory affected by active vasculitis based on FDG-PET uptake intensity between coronary and non-coronary arteritis patients at baseline study visit.

Arterial territory with active vasculitis	Total cohort N=130	Coronary arteritis N=12	No coronary arteritis N=118	P-value
R Carotid	31 (24%)	0 (0%)	31 (26%)	0.06
L Carotid	35 (27%)	0 (0%)	35 (30%)	0.02
Innominate	32 (25%)	3 (25%)	29 (25%)	1.00
R Subclavian	14 (11%)	1 (8%)	13 (11%)	1.00
L Subclavian	26 (20%)	2 (17%)	24 (21%)	1.00
Ascending Aorta	43 (33%)	7 (58%)	36 (31%)	0.06
Aortic Arch	48 (37%)	3 (25%)	45 (38%)	0.53
Descending Aorta	32 (25%)	3 (25%)	29 (25%)	1.00
Abdominal Aorta	34 (26%)	4 (33%)	30 (26%)	0.51
Pulmonary Artery	10 (8%)	0 (0%)	10 (9%)	0.60
R axillary Artery	5 (4%)	0 (0%)	5 (5%)	1.00
L axillary Artery	3 (2%)	0 (0%)	3 (3%)	1.00
R iliac Artery	6 (5%)	0 (0%)	6 (5%)	1.00
L iliac Artery	4 (3%)	0 (0%)	4 (4%)	1.00

At the baseline study visit, 4 of 13 (31%) patients with a history of coronary arteritis were clinically determined to have ongoing active vasculitis of the coronary arteries. All 4 of these patients had associated active vasculitis of the ascending aorta by FDG-PET (**Figure 3**). Two patients with a history of coronary arteritis had active vasculitis of the aorta by FDG-PET in the absence of active coronary arteritis. Across the entire cohort, the sensitivity of FDG-PET activity in the ascending aorta to predict active coronary arteritis was 100% (95% confidence interval 40-100%), and the specificity was 67% (95% confidence interval 60-77%).

## Discussion

This study is one of the most comprehensive efforts to date to describe coronary arteritis within an ongoing prospective observational cohort study of TAK and one of the only studies to evaluate standardized multimodal imaging to detail the role of cardiac imaging in the context of disease management. The determined prevalence of coronary arteritis in TAK was 9%, which confirms prior estimates. Coronary arteritis usually occurs at the time of diagnosis in TAK but can occur later into the disease course. The clinical manifestations of coronary arteritis range from asymptomatic disease that is incidentally detected by imaging to life-threatening myocardial ischemia. Vasculitic lesions of the coronary arteries in TAK are typically stenosing and involve the more proximal segments. Coronary aneurysms or distal disease should prompt additional diagnostic considerations. Favorable clinical outcomes are generally achievable but often require medical therapy and vascular intervention. Complications related to vascular grafts or stents are common and often prompt additional vascular procedures. Anti-cytokine medical therapies may be more effective than cytotoxic therapies.

Strong clinical associations or risk factors associated with coronary arteritis among patients with TAK were not observed. All patients with coronary arteritis were female; however, this may reflect the strong female predominance of the disease in general. Age at disease onset, age at diagnosis, and increased disease duration were not risk factors for coronary arteritis. Similarly, no obvious patterns of clinical symptoms or vascular involvement were associated with coronary arteritis. Previous studies have suggested an association between coronary involvement and Numano Type V (17), which is defined by diffuse arterial involvement throughout the thoracic and abdominal vasculature, but this pattern was only observed in 38% of patients with coronary arteritis in this study. These observations support the hypothesis that coronary arteritis is not associated with a distinct risk profile in TAK.

This study uniquely emphasizes the value of multimodal imaging assessment in patients with coronary arteritis. Because noninvasive angiography of the chest is not sufficient to visualize the coronary arteries, dedicated cardiac imaging is needed if coronary arteritis is clinically suspected. Cardiac CTA is useful to define the morphology of the coronary arteries and to assess patency of prior revascularization procedures. In conjunction, stress CMR imaging can be useful to clarify cardiac symptoms and identify areas of ongoing active ischemia, as well as infarction. FDG-PET is increasingly used to detect vascular inflammation in patients with large-vessel vasculitis (18). Non-specific cardiac FDG uptake in patients who do not adhere to a strict low-carbohydrate diet and suboptimal scanner resolution often prevents direct assessment of the coronary arteries by PET. However, given the majority of vasculitic lesions of the coronaries involve the ostia, significant FDG uptake in the ascending aorta may be associated with active coronary arteritis. In this study, although FDG uptake in the ascending aorta was not a specific marker for active coronary vasculitis across all patients with TAK, ascending aortitis in a patient with a history of known coronary involvement should prompt additional diagnostic consideration for coronary arteritis.

Coronary arteritis presents challenging management considerations in TAK. Because coronary arteritis often occurs at the time of initial disease presentation, diagnostic uncertainty can complicate management in the acute setting. In general, patients should be managed with medical therapy whenever possible, with vascular intervention reserved for situations where immediate reperfusion is medically necessary. While cyclophosphamide is often considered for patients with many different forms of systemic vasculitis in acute and organ-threatening clinical scenarios, the drug was not effective in the two patients with TAK who received cyclophosphamide for coronary arteritis in this cohort. In contrast, treatment with tocilizumab or a TNF inhibitor was highly effective and should be strongly considered in the initial management of these patients, without clear preference for one over the other.

Conventionally, bypass grafts have been favored over PCI with stents to manage coronary ischemia in TAK (19, 20); however, this study documented a similar rate of complications for either approach, with 50% of patients requiring additional vascular interventional procedures due to graft occlusion or in-stent restenosis. The decision to pursue grafting versus stenting should be informed by institutional capabilities and shared decision making with the patient. Reassuringly, all patients in this study eventually achieved medical stability even if multiple interventional procedures were necessary, consistent with findings from a large observational cohort study that looked at outcomes from vascular interventional procedures involving the peripheral vasculature in TAK (25).

This study has several potential limitations to consider. Dedicated cardiac imaging was performed only in patients with known or clinical suspected coronary arteritis, which may lead to underestimation of the prevalence of coronary involvement across the cohort. While accelerated atherosclerosis can occur in association with inflammatory disease in general, prevalence of atherosclerotic coronary disease was not assessed in this cohort. Although no strong clinical risk factors were observed in association with coronary arteritis in TAK, the small sample size reduces power to detect weaker clinical associations. No adjustment for multiple comparisons was performed, and the few differences reported in this study could be due to chance alone. Survival bias may have influenced the favorable long-term outcomes observed in the cohort.

Despite these limitations, this study has several noteworthy strengths. In particular, patients underwent comprehensive multimodal imaging at a single institution per standardized imaging protocols with central review of all imaging studies. Additionally, all patients were comprehensively assessed with use of standardized case report forms in an ongoing prospective observational cohort study with comprehensive review off all available outside records and imaging studies.

In conclusion, coronary arteritis is an uncommon but potentially life-threatening manifestation in TAK. Multimodal imaging can be useful to diagnose, monitor, and manage coronary arteritis in these patients. While medical therapy is preferred, vascular intervention may be necessary, but complications from attempts at vascular reperfusion are common. These data will be useful to inform future clinical guidelines in the management of patients with TAK.

## Data Availability

The original contributions presented in the study are included in the article/supplementary material. Further inquiries can be directed to the corresponding author.
